# DNA-PKcs plays a dominant role in the regulation of H2AX phosphorylation in response to DNA damage and cell cycle progression

**DOI:** 10.1186/1471-2199-11-18

**Published:** 2010-03-06

**Authors:** Jing An, Yue-Cheng Huang, Qing-Zhi Xu, Li-Jun Zhou, Zeng-Fu Shang, Bo Huang, Yu Wang, Xiao-Dan Liu, De-Chang Wu, Ping-Kun Zhou

**Affiliations:** 1 Department of Radiation Toxicology and Oncology, Beijing Institute of Radiation Medicine, Beijing 100850, PR China; 2 Institute of Environmental Pollution and Health, School of Environmental and Chemical Engineering, Shanghai University, Shanghai 200072, PR China; 3 The Center of Clinical Laboratory, Navy General Hospital, PLA, Beijing 100037, PR China

## Abstract

**Background:**

When DNA double-strand breaks (DSB) are induced by ionizing radiation (IR) in cells, histone H2AX is quickly phosphorylated into γ-H2AX (p-S139) around the DSB site. The necessity of DNA-PKcs in regulating the phosphorylation of H2AX in response to DNA damage and cell cycle progression was investigated.

**Results:**

The level of γH2AX in HeLa cells increased rapidly with a peak level at 0.25 - 1.0 h after 4 Gy γ irradiation. SiRNA-mediated depression of DNA-PKcs resulted in a strikingly decreased level of γH2AX. An increased γH2AX was also induced in the ATM deficient cell line AT5BIVA at 0.5 - 1.0 h after 4 Gy γ rays, and this IR-increased γH2AX in ATM deficient cells was dramatically abolished by the PIKK inhibitor wortmannin and the DNA-PKcs specific inhibitor NU7026. A high level of constitutive expression of γH2AX was observed in another ATM deficient cell line ATS4. The alteration of γH2AX level associated with cell cycle progression was also observed. HeLa cells with siRNA-depressed DNA-PKcs (HeLa-H1) or normal level DNA-PKcs (HeLa-NC) were synchronized at the G1 phase with the thymidine double-blocking method. At ~5 h after the synchronized cells were released from the G1 block, the S phase cells were dominant (80%) for both HeLa-H1 and HeLa-NC cells. At 8 - 9 h after the synchronized cells released from the G1 block, the proportion of G2/M population reached 56 - 60% for HeLa-NC cells, which was higher than that for HeLa H1 cells (33 - 40%). Consistently, the proportion of S phase for HeLa-NC cells decreased to ~15%; while a higher level (26 - 33%) was still maintained for the DNA-PKcs depleted HeLa-H1 cells during this period. In HeLa-NC cells, the γH2AX level increased gradually as the cells were released from the G1 block and entered the G2/M phase. However, this γH2AX alteration associated with cell cycle progressing was remarkably suppressed in the DNA-PKcs depleted HeLa-H1 cells, while wortmannin and NU7026 could also suppress this cell cycle related phosphorylation of H2AX. Furthermore, inhibition of GSK3β activity with LiCl or specific siRNA could up-regulate the γH2AX level and prolong the time of increased γH2AX to 10 h or more after 4 Gy. GSK3β is a negative regulation target of DNA-PKcs/Akt signaling via phosphorylation on Ser9, which leads to its inactivation. Depression of DNA-PKcs in HeLa cells leads to a decreased phosphorylation of Akt on Ser473 and its target GSK3β on Ser9, which, in other words, results in an increased activation of GSK3β. In addition, inhibition of PDK (another up-stream regulator of Akt/GSK3β) by siRNA can also decrease the induction of γH2AX in response to both DNA damage and cell cycle progression.

**Conclusion:**

DNA-PKcs plays a dominant role in regulating the phosphorylation of H2AX in response to both DNA damage and cell cycle progression. It can directly phosphorylate H2AX independent of ATM and indirectly modulate the phosphorylation level of γH2AX via the Akt/GSK3 β signal pathway.

## Background

The DNA double-strand break (DSB) is a major type of cellular damage induced by ionizing radiation. In mammalian cells, DSB in chromatin promptly initiates the phosphorylation of histone H2AX at Ser139 to generate γ-H2AX foci in megabase regions localized around each individual break, which is an essential and efficient coordinator of recognition and repair of DNA damage to ensure the maintenance of genomic stability [1-3]. The H2AX^-/- ^mouse displays multiple phenotypes, such as enhanced radiosensitivity, delayed growth and immune defects [4]. The H2AX gene, a member of the H2A family, is located at 11q23.2-q23.3, and encodes a 142 amino acid protein. In response to DNA damage, H2AX is phosphorylated in the C-terminus at Ser139, part of a consensus SQE (Ser-Gln-Glu) motif that is a common recognition site for phosphorylation by the phosphatidylinositol-3-OH-kinase-like family of protein kinases (PIKKs) [5,6]. Phosphorylation of H2AX can be mediated by all three major PIKK proteins, ATM [7,8], ATR [9,10] and DNA-dependent protein kinase [8,11]. H2AX phosphorylation by ATM, which is linked to the induction of DSBs, has been widely reported [7,12-14]. Phosphorylation of H2AX by ATR has been shown to occur in response to UV-induced DNA damage [9,15] or replication stress [10]. DNA-PKcs is the catalytic subunit of the DNA-PK complex, in which the Ku70 and Ku80 heterodimer binds to the ends of broken DNA, and DNA-PKcs is recruited to form the active kinase complex. DNA-PKcs was shown to be activated in nucleosomes via Ku binding to the ends of nucleosomal DNA. Activated DNA-PKcs is capable of phosphorylating H2AX within nucleosomes, and histone acetylation stimulates the phosphorylation of H2AX largely by DNA-PKcs [11]. DNA-PKcs has also been shown to phosphorylate H2AX during apoptotic DNA fragmentation [16,17], and is responsible for the enhanced phosphorylation of H2AX under hypertonic conditions [18]. A direct connection between γH2AX foci formation and disappearance, and DNA-PK activity, has also been demonstrated in response to replication stress induced by low levels of the replication inhibitor aphidicolin [19] and replication-associated DNA damage [20]. In addition, H2AX phosphorylation was also found to be dependent on DNA-PK in the DNA damage response induced by the death receptor ligand TRAIL [17]. As all three PIKK members are able to phosphorylate H2AX, it is important to understand which plays the dominant role in phosphorylating H2AX in response to gamma radiation-induced DNA damage. Burma *et al*. previously reported that H2AX phosphorylation at Ser139 after X-irradiation is ATM-dependent in mouse embryonic fibroblasts [7], while Stiff *et al*. showed that both ATM and DNA-PK can promote radiation-induced phosphorylation of H2AX in human and rodent cells [13]. Koike *et al*. have recently reported that the regulatory mechanism of γH2AX generation is tissue-specific in mice, and that H2AX phosphorylation at Ser139 in the spleen after X-irradiation is mainly mediated by DNA-PK [21]. Therefore, further investigation is needed to elucidate the coordination of ATM and DNA-PKcs in regulating H2AX phosphorylation in response to different stimuli or conditions.

In addition, γH2AX dephosphorylation is another mechanism by which cellular levels of γH2AX may be regulated, and dephosphorylation of γH2AX is also important for completing the DNA repair process. In order to restore chromatin integrity and structure after the repair process, phosphorylated H2AX must be converted to H2AX, which is in principle accomplished either by replacing γH2AX in the nucleosome with H2AX, or by dephosphorylating nucleosomal γH2AX directly. In mammalian cells, the phosphatases PP2A and PP4 have been shown to be involved in the dephosphorylation of γH2AX [22-24].

Using siRNA-mediated depletion of DNA-PKcs, ATM-deficient cells, the PI3K kinase inhibitor wortmannin, and the specific inhibitor of DNA-PKcs NU7026, we now show that DNA-PK plays a crucial role for H2AX phosphorylation in response to ionizing radiation and during cell cycle progression.

The AGC family Ser/Thr kinase protein kinase B (PKB/Akt)/GSK3β is a common downstream signaling pathway of both DNA-PKcs and 3-phosphoinositide-dependent kinase 1 (PDK 1), in which Akt is fully activated through phosphorylation of two key residues, Thr308 by PDK1 [25] and Ser473 by DNA-PKcs [26] in response to DNA damage. The phosphorylated Akt can then inactivate GSK3β by phosphorylating it on Ser9. Therefore, we have further determined whether Akt/GSK3β signaling involves in the regulation of H2AX phosphorylation. We found that inactivation of GSK3β using LiCl or specific siRNA, can upregulate γH2AX levels and block the decrease in γH2AX caused by loss of DNA-PKcs. SiRNA-mediated inhibition of PDK 1 can also downregulate the levels of γH2AX. Our results demonstrate that DNA-PKcs is a critical kinase, which regulates the level of phosphorylated H2AX independent of ATM.

## Results

### DNA-PKcs is required for H2AX phosphorylation in response to DNA damage induced by ionizing radiation

**Figure 1:**
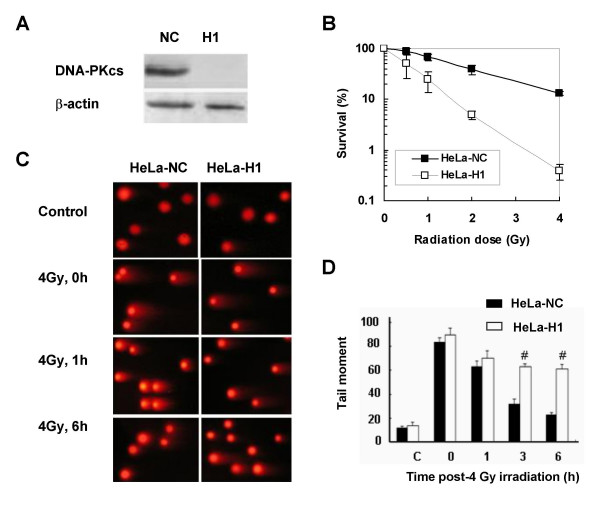
**Depletion of DNA-PKcs inhibits DNA double-strand break (DSB) repair and sensitizes HeLa cells to ionizing radiation**. A: Depletion of DNA-PKcs by specific siRNA in HeLa-H1 cells. B: Survival curves of DNA-PKcs-depleted cells (HeLa-H1) and control cells (HeLa-NC). C: Comet images of DNA DSB detected by neutral single cell gel electrophoresis 0-6 h post-irradiation. D: The repair kinetics of 4 Gy-induced DNA DSBs detected by comet assay. The tail moment was used as the endpoint of DNA DSB. Each bar represents the mean tail moment from three independent experiments. 100 individual comets were counted per time point for each experiment. ^# ^*p *< 0.01, as compared to the HeLa-NC cells at the same time point.

**Figure 2:**
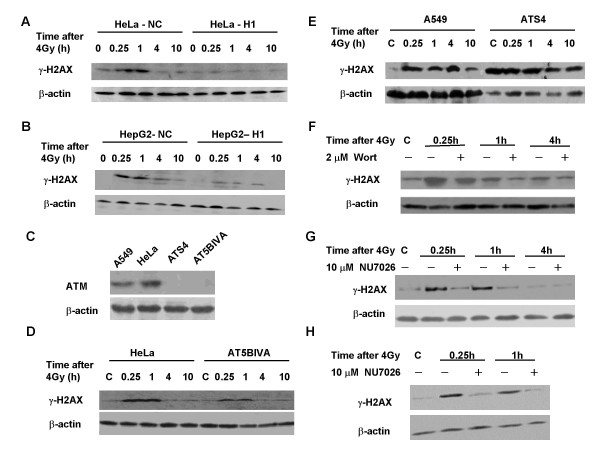
**Phosphorylation of H2AX in response to radiation-induced DNA damage in the presence or absence of DNA-PKcs and ATM**. A, B: Phosphorylated H2AX (γH2AX) levels in DNA-PKcs depleted HeLa-H1 (A), HepG2-H1 [39] (B) cells and the control HeLa-NC or HepG2-NC cells. Cells were harvested at 0, 0.25, 1, 4 and 10 h after 4 Gy γ-irradiation. Protein expression was assayed by Western blotting. C: Expression of phospho-ATM at Ser-1981 detected at 0.5 h after 4 Gy γ-irradiation. D, E: H2AX phosphorylation in ATM-deficient cells AT5BIVA (D) and ATS4 (E) after 4 Gy γ-irradiation. Cells were harvested at 0, 0.25, 1, 4 and 10 h post-irradiation, and protein expression was assayed by Western blotting. F, G: The effect of the PI3K inhibitor wortmannin (F) and the DNA-PKcs specific inhibitor NU7026 (G) on H2AX phosphorylation in AT5BIVA cells after γ-irradiation. AT5BIVA cells were pretreated with 2 μM wortmannin or 10 μM NU7026 for 2 h, then irradiated with 4 Gy. The cells were harvested at 0, 0.25, 1 and 4 h after irradiation. Protein expression was assayed by Western blotting. H: The effect of NU7026 treatment (10 μM) on H2AX phosphorylation in HeLa-NC cells after 4 Gy irradiation.

A stable cell line, in which DNA-PKcs expression was downregulated via siRNA (HeLa-H1), was generated from HeLa cells (Figure 1A). Depletion of DNA-PKcs resulted in an increased sensitivity to radiation (Figure 1B) and decreased repair of DNA double-strand breaks (DSB) (Figure 1C and 1D). Comet assays showed that 80% of IR-induced DSB, as measured by the tail moment, were repaired at 3 h after 4 Gy in the DNA-PKcs-expressing HeLa-NC cells, compared to only 30% DSB repair in HeLa-H1 cells. After 4 Gy, the level of γH2AX in HeLa-NC cells was quickly elevated, reaching a peak at about 1 h post-irradiation. In contrast, the level of γH2AX was barely increased in DNA-PKcs-silenced HeLa-H1 cells (Figure 2A), indicating that DNA-PKcs is necessary for the phosphorylation of H2AX in response to radiation. In order to confirm this result, we detected the γH2AX level in HepG2-H1, another DNA-PKcs depleted cell line generated from HepG2 cells transfected with a specific siRNA constructs targeting the DNA-PKcs catalytic motif (nucleotides 11637~11655, H1). It was found that the induction of γH2AX by 4 Gy in HepG2-H1 cells was also much lower than that in control HepG2-NC cells (Figure 2B).

To determine the relative contributions of ATM and DNA-PKcs to the phosphorylation of H2AX in response to DNA damage, we measured changes in γH2AX levels in two ATM-deficient cell lines, AT5BIVA and ATS4 after 4 Gy γ-irradiation (Figure 2C). An increased autophosphorylated DNA-PKcs on Ser2056 was induced at least 15 to 60 min after 4 Gy in these two cell lines (data not shown). As shown in Figure 2D, a clear increase in γH2AX was induced in AT5BIVA cells at 0.25-1 h after γ-irradiation, although the amount of increased γH2AX level was slightly less than that of HeLa cells. Unexpectedly, the ATM-deficient cell line ATS4 showed a high constitutive level of γH2AX (Figure 2E). Indeed, the level of γH2AX in ATS4 cells under normal growing conditions was so high that radiation did not induce any further increase. Wortmannin has inhibitory activity against both ATM and DNA-PK, and it effectively reduced the induction of H2AX phosphorylation by radiation in AT5BIVA cells (Figure 2F). To more specifically investigate the role of DNA-PK in radiation-induced H2AX phosphorylation, we used the competitive and highly specific DNA-PK inhibitor NU7026. Treatment of cells with 10 μM NU7026 largely abolished the radiation-induced phosphorylation of H2AX in AT5BIVA cells (Figure 2G) as well as ATM efficient HeLa-NC cells (Figure 2H). These data indicate that DNA-PKcs plays a critical role in the phosphorylation of H2AX in response to DNA damage.

### DNA-PKcs is required for H2AX phosphorylation associated with cell cycle progression

**Figure 3:**
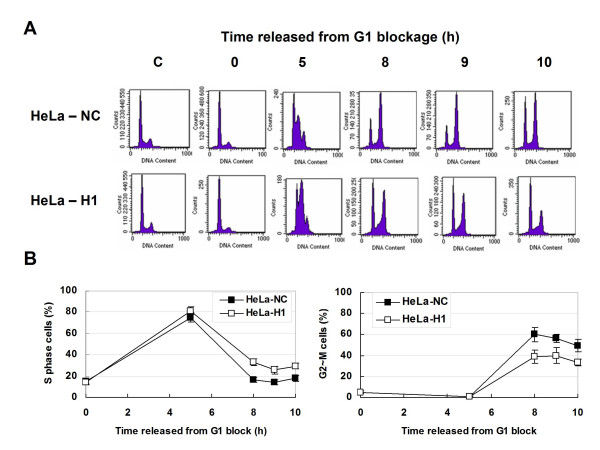
**Cell cycle progression of the synchronized DNA-PKcs-depressed HeLa-H1 cells and control HeLa-NC**. A: Representative histograms of flow cytometry analysis. The cells synchronized in G1 phase by TdR double-blocking. G1 arrested cells were cultured in fresh DMEM, collected at 0, 5, 8, 9, 10 h after released from G1 block, analyzed by flow cytometry. B: Quantitative data of the cell cycle distribution of the cells released from G1 blockage by TdR double-blocking. Left panel, the proportion of S-phase cells at different times after released from G1 block. Right panel, the proportion of G2/M-phase cells at different times after cell cycle re-entry.

To synchronize cells, thymidine was used to block cells at the G1/S transition by reversibly inhibiting DNA synthesis, without affecting the progression of other cell cycle phase. Different phases of synchronized cells were obtained by harvesting cells at different times after release from a thymidine-induced G1 block (Figure 3A and 3B). As shown in Figure 3B (left), the majority of the cell population was in S-phase (80%) at 5 h and proceeded into G2/M phase 8-10 h after release from the G1 block in both HeLa-H1 and HeLa-NC cells. However, the S-phase population of HeLa-H1 cells at 8-10 h after release (26-33%) was higher than that of HeLa-NC cells (~15%). Consistently, the G2/M-phase proportion of HeLa-H1 cells (33-40%) was relatively lower than that of HeLa-NC cells (56-60%) (Figure 3B-right).

**Figure 4:**
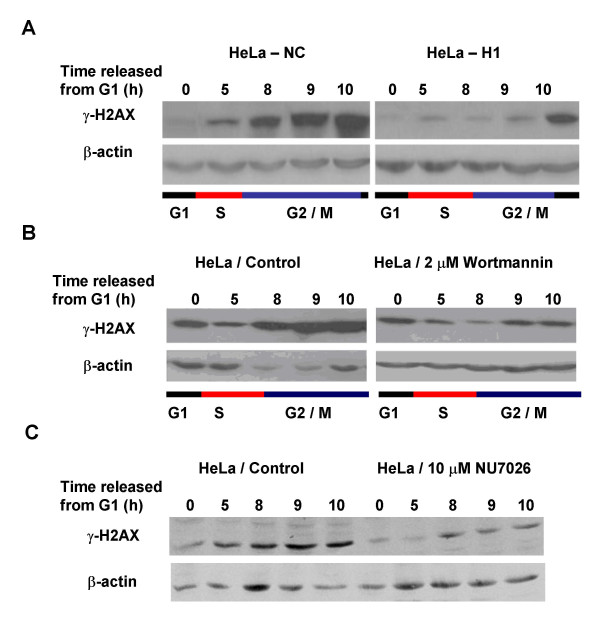
**Phosphorylation of H2AX associated with cell cycle progression and the relative contributions of DNA-PKcs**. A: Levels of γH2AX associated with cell cycle progression in DNA-PKcs-depleted HeLa-H1 (right) and control HeLa-NC cells (left). B: The effect of wortmannin on cell-cycle associated H2AX phosphorylation. After G1 synchronization by TdR double-blocking, cells were cultured in fresh DMEM supplemented with 2 μM wortmannin, then collected at 0, 5, 8, 9, 10 h, and analyzed by Western blotting. C: The effect of NU2076 on cell-cycle associated H2AX phosphorylation. After G1 synchronization by TdR double-blocking, cells were cultured in fresh DMEM supplemented with 10 μM NU2076, then collected at 0, 5, 8, 9, 10 h, and analyzed by Western blotting.

**Figure 5:**
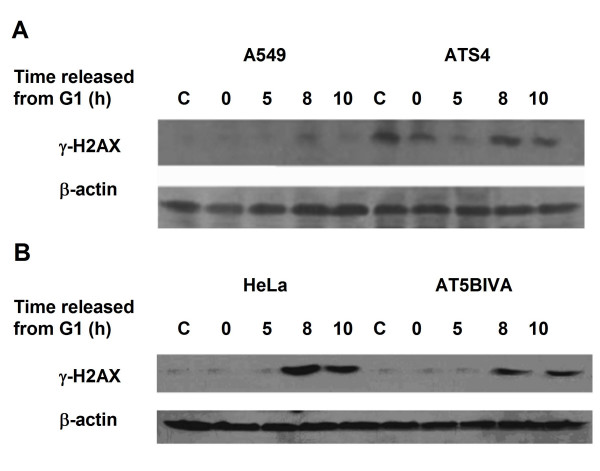
**Cell Cycle associated H2AX phosphorylation in ATM deficient cells**. The levels of phosphorylated H2AX at different times after release from G1 block were detected in ATM deficient cell lines ATS4 (A) and AT5BIVA (B). Synchronized G1 cells were cultured in fresh DMEM, harvested at 0, 5, 8 and 10 h after cell cycle re-entry, and protein expression was assayed by Western blotting.

An alteration of phosphorylated H2AX level associated with cell cycle progression has been observed (Figure 4A). In HeLa-NC cells, γH2AX levels gradually increased 5 h after release from the G1 block, and reached a peak at 8-10 h, i.e. with the majority of cells in G2/M-phase. This cell cycle associated increase of γH2AX levels in the DNA-PKcs-depleted HeLa-H1 cells was much lower than that in control HeLa-NC cells. The expression peak in HeLa-H1 cells was delayed to 10 h after the G1 release and was also much weaker than in HeLa-NC cells. Treatment with 2 μM wortmannin inhibited the cell cycle-associated phosphorylation of H2AX (Figure 4B). Moreover, the DNA-PKcs specific inhibitor NU7026 also largely abolished this cell cycle-associated phosphorylation of H2AX (Figure 4C). Therefore, we conclude that DNA-PKcs is required for the phosphorylation of H2AX associated with cell cycle progression. In order to determine whether ATM is necessary for the cell cycle-associated phosphorylation of H2AX, we examined γH2AX levels in synchronized ATM-deficient cells. An increase in γH2AX was clearly seen at 8-10 h after release from the G1 block for two ATM-deficient cell lines, ATS4 (Figure 5A) and AT5BIVA (Figure 5B).

### The Akt/GSK3β signaling pathway is involved in the regulation of H2AX phosphorylation

**Figure 6:**
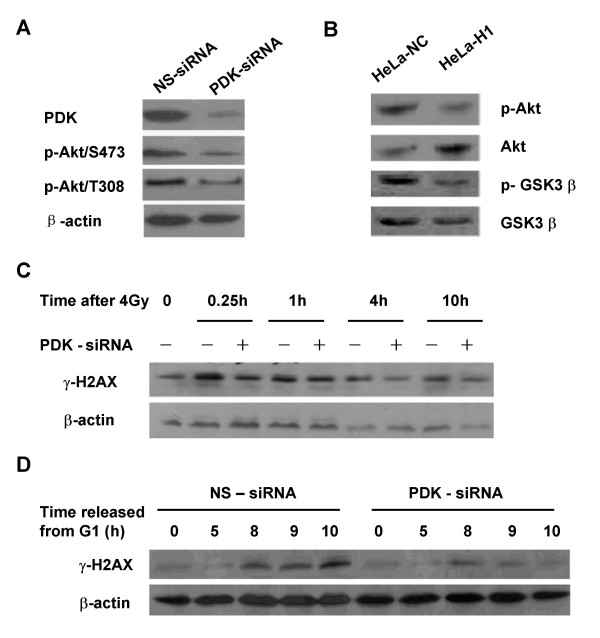
**Regulation of phosphoinositide-dependent kinase (PDK) on the phosphorylation of H2AX**. A: RNAi mediated depletion of PDK protein. HeLa cells were transfected with 50 nM PDK specific siRNA molecules or non-specific (ns) control siRNA. Western blotting shows PDK expression. B: Phosphorylation of Akt at Ser473 and GSK3β at Ser9 was decreased in the DNA-PKcs depleted HeLa-H1 cells compared to control HeLa-NC cells. C: PDK regulates the phosphorylation of H2AX in response to DNA damage induced by 4 Gy of γ-irradiation. After 48 h incubation with 50 nM PDK-specific siRNA or non-specific (ns) control siRNA, cells were irradiated with 4 Gy γ rays, then harvested 0, 0.5, 1, 4, 10 h post-irradiation and analyzed by Western blotting. D: PDK regulates the phosphorylation of H2AX associated with cell cycle progression. After 24 h incubation with 50 nM PDK-specific siRNA or non-specific (ns) control siRNA, cells were synchronized in G1 phase by TdR double-blocking, then released and harvested after 5 h, for S-phase, and at 8, 9, and 10 h, for G2/M phase. The culture medium was supplemented with 50 nM siRNA molecules during the period of synchronization and cell cycle progression. Protein expression was assayed by Western blotting.

**Figure 7:**
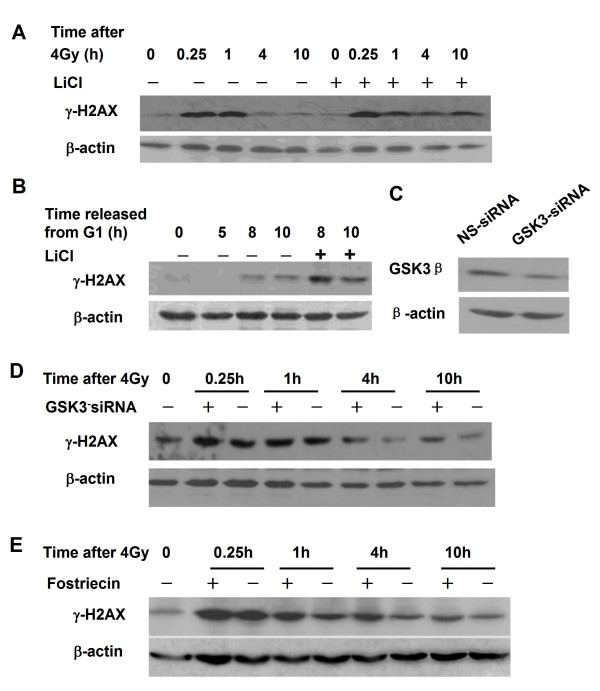
**Regulation of H2AX phosphorylation in response to DNA damage and cell cycle progression by GSK3β**. A: The GSK3β inhibitor LiCl prolongs phosphorylated H2AX increase in response to 4 Gy irradiation. HeLa cells were pretreated with 40 μM LiCl for 2 h, irradiated with 4 Gy γ rays and harvested at 0, 0.5, 1, 4 and 10 h after irradiation. Protein expression was assayed by Western blotting. B: GSK3β inhibitor LiCl enhanced the phosphorylation of H2AX in G2/M phase cells. To release the cells from G1 block and inhibit GSK3β activity, synchronized HeLa cells were grown in DMEM medium supplemented with 40 μM LiCl. S-phase cells were harvested at 5 h and G2/M phase cells at 8, 9 and10 h after released. Protein expression was assayed by Western blotting. C: RNAi depletion of GSK3β. HeLa cells were transfected with 50 nM GSK3β siRNA or non-specific (ns) control siRNA molecules. GSK3β expression was determined by Western blotting. D: Effect of GSK3β depletion on the phosphorylation of H2AX induced by 4 Gy γ-irradiation. After 48 h incubation with 50 nM GSK3β-specific siRNA or control non-specific (ns), cells were irradiated with 4 Gy γ rays, and harvested at 0, 0.25, 1, 4 and 10 h post-irradiation. Protein expression was assayed by Western blotting. E: Effect of the PP2A inhibitor fostriecin on H2AX phosphorylation. HeLa cells were pretreated with 50 nM fostriecin for 2 h, and then irradiated with 4 Gy γ rays, and harvested at 0, 0.25, 1, 4 and 10 h post-irradiation. Protein expression was assayed by Western blotting.

To explore the potential alternative mechanism by which DNA-PKcs regulates H2AX phosphorylation, we investigated the role of Akt and GSK3β, downstream targets of both DNA-PKcs and phosphoinositide-dependent kinase 1 (PDK 1), in the regulation of γH2AX. Indeed, depletion of PDK by RNAi led to not only a decrease in phosphorylated Akt on Thr308 and Ser473 (Figure 6A), but also inhibition on the phosphorylation of H2AX in response to 4 Gy (Figure 6C) and in G2/M-phase cells (Figure 6D). These results indicate that Akt/GSK3β signaling could play an important role in regulating the phosphorylation of H2AX. As shown in Figure 6B, siRNA-mediated downregulation of DNA-PKcs led to a decrease in the phosphorylation of Akt at Ser473, and of GSK3β at Ser9, as well as activation of GSK3β which is negatively regulated by Akt. Treatment with 40 μM LiCl, an inhibitor of GSK3β, prolonged the duration of increased γH2AX levels to at least 10 h post-irradiation (Figure 7A). LiCl treatment also increased γH2AX levels in the thymidine-synchronized HeLa cells in G2/M phase, i.e. 8-10 h after release from G1 block (Figure 7B). Moreover, downregulation of GSK3β expression by specific GSK3β siRNA (Figure 7C) also significantly enhanced and prolonged the phosphorylation of H2AX in response to irradiation (Figure 7D) and in G2/M phase cells (data not shown). It has been reported that phophatase PP2A and PP4 can dephosphorylate γH2AX [22-24]. We demonstrated that the increased γH2AX induced by GSK3β inhibitor or specific siRNA molecules is equivalent to that induced by the PP2A inhibitor fostriecin (Figure 7E). Taken together, these data suggest that the Akt/GSK3β signaling pathway could also be involved in regulating the dephosphorylation of γH2AX.

## Discussion

DNA DSB induced by gamma irradiation, or other DSB inducers, leads to rapid phosphorylation of H2AX at Ser139 by ATM, ATR and DNA-PKcs, resulting in γH2AX [3]. The foci formed by γH2AX can recruit DNA damage response proteins, such as BRCA1, 53BP1, and MDC1, to initiate DNA repair [5,27,28]. In addition, other related proteins such as NBS, CHK2, SMC1, Rad51 and Rad50, are also co-localized to γH2AX foci, but these proteins may be indirectly induced by other DNA damage response proteins [29]. After γH2AX marks sites of DNA damage, the damage recruits repair proteins and dephosphorylation of γH2AX is necessary for release of repair proteins from the damage sites to complete the DNA repair process. The protein phosphatases 2A (PP2A) and PP4 were previously shown to be responsible for dephosphorylation of γH2AX [22-24]. It has been debated as to whether Ser139 of H2AX is phosphorylated by ATM alone or both ATM and DNA-PK after ionizing radiation [21]. Our data indicate that 4 Gy irradiation can rapidly induce H2AX phosphorylation (within 15 min), and that γH2AX then declines to normal levels after 4 h in HeLa cells. Depletion of DNA-PKcs by siRNA significantly abolished the IR-induced phosphorylation of H2AX in HeLa-H1 cells.

It was reported that deficiency of DNA-PKcs causes downregulation of ATM in HeLa cells [30]. Consistent with this, the ATM protein level was also decreased in HeLa-H1 cells in concordance with the reduced DNA-PKcs (data not shown), and this further contributes to the dramatic reduction of γH2AX in HeLa-H1 cells. A dramatic decrease of IR-induced γH2AX was also demonstrated in the DNA-PKcs-depleted HepG2-H1 cells (Figure 2B). In contrast, a dramatic increase in γH2AX was induced by radiation in ATM-deficient AT5BIVA and ATS4 cells (Figure 2D, E). Moreover, ATS4 cells had a very high constitutive level of γH2AX (Figure 2E). In addition, the DNA-PKcs-specific inhibitor NU7026 can effectively abolish IR-induced phosphorylation of H2AX.

Koike et al recently reported that the level of γH2AX also increased in mice lacking either ATM or DNA-PK following X-irradiation [21], and they suggested that the phosphorylation of H2AX and the elimination of γH2AX following radiation proceeds in both DNA-PK-dependent and independent manner in vivo. Furthermore, they also demonstrated a tissue-specific mechanism of γH2AX level regulation, e.g. the phosphorylation of H2AX at Ser139 after X-irradiation in the spleen is mainly mediated by the DNA-PK [21]. Low doses of replication-inhibitor aphidicolin (APH) induce DSBs in replicating cells, and the formation of these DSBs requires Bloom's syndrome-associated (BLM) helicase and Mus81 nuclease [31]. These APH-induced BLM and Mus81-dependent DSBs activate the phosphorylation of H2AX by ATR kinase, while the DSBs are transient and appear to be rapidly repaired by DNA-PK-dependent non-homologous end joining (NHEJ) [19]. Therefore, it could be more complicated than generally considered regarding the regulatory mechanisms of γH2AX levels after DNA damage induced by ionizing radiation. Our findings further suggest that DNA-PKcs plays no less important role than does ATM, or both are functionally complementary to each other, in modulating of H2AX phosphorylation in response to DNA damage induced by ionizing radiation. Phosphorylated H2AX has also been reported in untreated normal and tumor cells, which can be explained as the consequence of a physiological event that involve DNA recombination [12] or due to DNA damage induced by reactive oxygen species (ROS) generated by metabolic activity during progression through the cell cycle [32]. The increased constitutive γH2AX seen in ATS4 cells could reflect the high levels of residual DNA damage.

Information regarding the association of H2AX phosphorylation and cell cycle progression is scarce. One report showed ATR-mediated phosphorylation of H2AX generated during DNA replication [10]. Kurise *et al*. reported that blocking HL-60 cells at the G1/S transition by treatment with inhibitors of DNA replication (thymidine, aphidicolin and hydroxyurea) resulted in H2AX phosphorylation at Ser139, and that this effect is most pronounced in S-phase cells and in cells undergoing induced apoptosis [33]. In this study, we found that a peak of H2AX phosphorylation appeared when synchronized HeLa cells entered the G2/M phases, but not in G1-arrested cells or in cells during S-phase (Figure 4A). These results are consistent with a previous report by Ichijima *et al *[34]. ATM deficiency did not significantly affect cell cycle progression-associated H2AX phosphorylation (Figure 5A and 5B), but loss of DNA-PKcs by the siRNA strategy (Figure 4A) or chemical inhibitors (Figure 4B and 5C) led to a marked decrease in γH2AX levels. Therefore, we conclude that DNA-PKcs also plays a key role in regulating H2AX phosphorylation associated with cell cycle progression. In addition, DNA-PKcs depletion can lead to some delay in G2-phase entry (Figure 3A and 3B).

The AGC family Ser/Thr kinase protein kinase B (PKB/Akt) was originally identified to be a central regulator of cell metabolism, survival, and proliferation. Following mitogen stimulation, Akt is fully activated through phosphorylation of two key residues, Thr308 in the activation loop and Ser473 in the C-terminal hydrophobic motif. Akt/Thr308 is phosphorylated by 3-phosphoinositide-dependent kinase 1 (PDK1) [35], while Akt/Ser473 is a target of the mammalian target of rapamycin complex 2 (mTORC2) [36]. Akt/Ser473 has been also identified as one of the downstream substrates of DNA-PKcs in response to DNA damage [26], and the phosphorylated Akt can inactivate GSK3β by phosphorylating it on Ser9. The direct phosphorylation of Akt on S473 by DNA-PK requires a specific recognition sequence in the C-terminal hydrophobic motif surrounding the Ser-473 phospho-acceptor site in PKB [37]. Bozulic et al have recently shown that PDK1 is responsible for Akt/Thr308 phosphorylation in the DNA damage induced by ionizing radiation, and GSK3 phosphorylation after DNA damage depends on both DNA-PK-mediated and PDK1-mediated activation of Akt [25]. In PDK1^-/- ^cells, phosphorylation of Akt/Thr308 was not detected after ionizing radiation, and phosphorylation of Akt/Ser473 was also much lower than in wild-type cells. This was further reflected by the lack of phosphorylation of GSK3α although DNA-PKcs was active in PDK1^-/- ^cells. Our data demonstrate that inhibition of GSK3β by siRNA or LiCl resulted in increased phosphorylation of H2AX in G2/M phase cells (Figure 7B) and after irradiation (Figure 7A, D). Inhibition of GSK3β prolonged the time of γH2AX elevation after radiation, and which implicates GSK3β in promoting dephosphorylation of γH2AX. Therefore, we suggest another pathway of DNA-PKcs affecting the H2AX phosphorylation level, i.e., DNA-PKcs activates Akt via phosphorylation on Ser473, which in turn inactivates GSK3β via phosphorylating Ser9 [38]. The inactivated GSK3β loses its effect of negatively modulating the γH2AX level. Furthermore, depletion of PDK 1, an upstream regulator of Akt, by RNAi also results in a decrease in radiation-induced (Figure 6C) and cell cycle associated (Figure 6D) phosphorylation of H2AX, This further supports the involvement of Akt/GSK3β in regulating the γH2AX level.

## Conclusion

The phosphorylation and dephosphorylation of H2AX is necessary for the DNA damage repair process. In mammalian cells, there are likely multiple pathways involved in modulating γH2AX in response to DNA double-strand breaks (DSB) induced by ionizing radiation. Our results further demonstrate that DNA-PKcs plays a critical role in the phosphorylation of H2AX in response to both DNA damage and G2/M phase entry. DNA-PKcs can both directly phosphorylate H2AX and indirectly modulate the γH2AX level through the Akt/GSK3β signaling pathway. More studies are needed to elucidate the detailed mechanism of GSK3β regulating the γH2AX level in response to the DNA damage and during cell cycle progression.

## Methods

### Cell Culture and siRNA Transfection

HeLa, A549, ATS4, and AT5BIVA cells were maintained in Dulbecco's modified Eagle's medium (DMEM) containing 10% fetal bovine serum, 100 U/ml of penicillin and 100 μg/ml of streptomycin in a humidified chamber at 37°C in 5% CO_2_. HeLa-H1, HeLa-NC, HeG2-H1 and HepG2-NC cells were generated from HeLa cells or HepG2 cells via stable transfection with specific siRNA constructs targeting the DNA-PKcs catalytic motif (nucleotides 11637~11655, H1), or a control construct (NC), respectively [39].

The siRNA duplexes used in this study were synthesized by Genechem (Shanghai, China), including PDK-specific siRNA (sense strand: 5'-CAACAUAGAGCAGUACAUU-3'), GSK3β-specific siRNA (sense strand: 5'-GAGCAAAUCAGAGAAAUGAdtdt-3'), and non-specific control siRNA (sense strand: 5'-UUCUCCGAACGUGUCACGUdtdt-3'). For siRNA transfection, 2.5 × 10^5 ^cells/well were plated in 6-well culture plates. After 24 h, 30 μl of Lipofectamine 2000 reagent (Invitrogen, Carlsbad, CA) was added to 1.5 ml DMEM without antibiotics and serum, and incubated at room temperature for 5 min (solution A). 50 nM siRNA was added to 1.5 ml DMEM without antibiotics and serum (solution B). Solution A and solution B were then mixed and incubated at room temperature for 20 min. The cell culture medium was removed, and then 0.5 ml of the Lipofectamine 2000-siRNA mixture and 1.5 ml of fresh DMEM without antibiotics were added to each culture well and gently mixed. After 48 hours of incubation, the cells were harvested for western blot analysis.

### Irradiation

Cells were irradiated at room temperature using a cobalt-60 γ-ray source at a dose rate of 1.7 Gy min^-1^. For the mock-radiation control, cell cultures were placed in a non-radiation room with the same environment for the same amount of time as treated cultures.

### Chemicals and Antibodies

Wortmannin and NU7026 were purchased from Sigma-Aldrich. All antibodies were purchased commercially: anti-β-actin (I-19-R, Santa Cruz, CA), anti-DNA-PKcs (H-163, Santa Cruz, CA), γH2AX (05-636, Upstate Biotechnology, Charlottesville, VA), anti-GSK3β (#9332, Cell Signaling, Danvers, MA), anti-phospho-GSK3β (Ser-9, #9336, Cell Signaling, Danvers, MA), anti-PDK1 (#3062, Cell signaling, Danvers, MA), anti-phospho-Akt (Ser-473, #9271, Cell Signaling, Danvers, MA), anti-phospho-ATM (Ser-1981, Cell Signaling, Danvers, MA), anti-rabbit IgG(H+L)/HRP (ZB-2301, Zhongshan, Beijing, China), and anti-mouse IgG(H+L)/HRP (ZB-2305, Zhongshan, Beijing, China).

### Comet assay

After irradiation with 4 Gy γ-rays and culture for 0-6 h, cells were collected and mixed with low melting point (LMP) agarose at 37°C. This mixture was placed on top of a previous previously formed layer of 0.5% normal melting point agarose on a slide, covered with a cover slip, and returned to 4°C until solidified. Then, the cover slip was gently removed and another layer of normal melting point agarose was added on top. The slide was again covered with a cover slip and placed at 4°C until the mixture solidified. The slide was placed in chilled neutral lysis solution and subjected to electrophoresis. Thereafter, slides were gently washed with neutralization buffer, stained with ethidium bromide, and visualized under a fluorescence microscope. DNA damage was expressed as the tail moment, combining comet tail length and the proportion of DNA migrating into the tail.

### Cell synchronization using the thymidine double-blocking method

10^6 ^of cells were plated in 60 mm Petri dishes, and thymidine was added to a final concentration of 2 mM after cell adherence (about 6-8 h). The cells were cultured for 16 h. After removal of the thymidine and incubation for 10 h in the fresh DMEM solution, thymidine was added to a final concentration of 2 mM for an additional 16 h. After removal of thymidine again, synchronized cells were cultured in fresh DMEM and collected at different times for cell cycle analysis and western blotting.

### Cell cycle analysis using flow cytometry

The thymidine-synchronized cells were collected at different times after release from a G1 block. After washing twice with PBS solution, cells were fixed with chilled 70% alcohol at -20°C for 24 h. The cell sediment was collected by centrifugation (1,000 rpm, 3 min), washed twice with PBS solution, incubated with 20 μl RNase A (20 mg/ml) for 30 min at 37°C, and stained with 25 μg ml^-1 ^PI (Sigma) for 30 min at room temperature. The cell cycle distribution was then evaluated using flow cytometry. All experiments were repeated three times.

### Western blotting analysis

The cells were harvested and washed twice in ice-cold phosphate-buffered saline. Cell pellets were treated with lysis buffer (50 mmol/L Tris-HCL, pH7.5, 1% NP-40, 0.5% sodium deoxycholate, 150 mmol/L NaCl, with 1 protease inhibitor cocktail tablet per 50 ml solution), and the total protein was isolated. Protein (50 μg) was resolved on 8% SDS-PAGE gels and transferred onto the polyvinylidene difluoride (PVDF) membrane for immunoblotting. ECL was used for detection.

## Competing interests

The authors declare that they have no competing interests.

## Authors' contributions

JA carried out most of the study and participated in its design. YCH participated in cell cycle synchronization and flow cytometry analysis. QZX participated the work design and data discussion. LJZ participated in the DNA damage analysis. HB carried out the immunoblotting assays of the AT cell lines. YW participated in the establishment of DNA-PKcs depleted HeLa-H1/-H3 cells. XDL contributed to the cell culture work. DCW jointly conceived of the study and coordination. PKZ jointly conceived of the study, and coordination, participated in its design and drafted the manuscript. All authors read and approved the final manuscript.
